# Protocol for identification of cell-surface proteins with horseradish peroxidase

**DOI:** 10.1016/j.xpro.2025.104255

**Published:** 2025-12-11

**Authors:** Ling Wu, Vijaya Pandey, James A. Wohlschlegel, Baljit S. Khakh

**Affiliations:** 1 Department of Physiology, David Geffen School of Medicine, University of California, Los Angeles, Los Angeles, CA, USA; 2 Department of Neurobiology, David Geffen School of Medicine, University of California, Los Angeles, Los Angeles, CA, USA; 3 Department of Biological Chemistry, David Geffen School of Medicine, University of California, Los Angeles, Los Angeles, CA, USA

**Keywords:** molecular biology, neuroscience, molecular/chemical probes, protein biochemistry, proteomics, mass spectrometry

## Abstract

Cell-surface proteins (CSPs) play a central role in intercellular communication. Here, we outline a protocol for the cell-type-specific proteomic profiling of CSPs in mice based on horseradish peroxidase (HRP). We describe steps for adeno-associated virus (AAV) injection, biotinylation reaction, tissue homogenization, streptavidin pull-down, and mass spectrometry characterization, followed by quality control and data interpretation. This approach enables systematic identification of CSPs underlying cell-cell interactions in health and disease.

For complete details on the use and execution of this protocol, please refer to Wu et al.^1^

## Highlights

• HRP-based proximity labeling approach to identify cell-surface proteins in mouse brain cells
• A step-by-step HRP protocol including experimental design and protein identification
• Guidance on data processing and interpretation

## Abstract (editor highlights)

Publisher’s note: Undertaking any experimental protocol requires adherence to local institutional guidelines for laboratory safety and ethics.

## Graphical abstract

**Figure:**
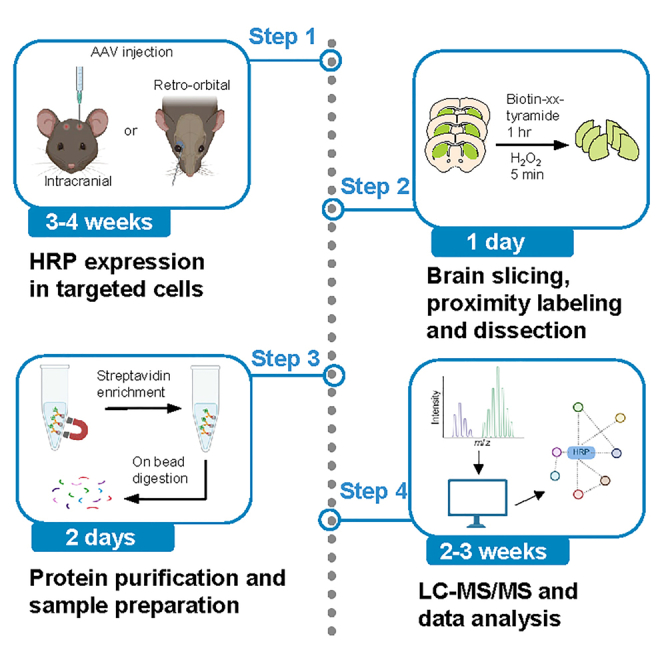

## Before you begin

Astrocytes closely contact neurons and other cell types through complex membrane structures within their bushy morphologies.^2^ Molecular interactions at these cell–cell interfaces, mediated by CSPs, are essential for astrocytes to communicate with neighboring cells, particularly neurons, and to carry out their diverse functions.^3^^,^^4^^,^^5^ Recently advances in proximity-labeling based proteomics have enabled the profiling of CSPs with both cell-type specificity and spatial resolution.^6^^,^^7^^,^^8^ By genetically targeting horseradish peroxidase (HRP) to the cell-surface of striatal astrocytes, where the enzyme generates reactive radicals to covalently tag neighboring proteins with biotin that are in turn characterized by LC-MS/MS, we obtain rich putative cell-surface proteomes of striatal astrocytes.

The protocol below describes specific steps for proteomic profiling of CSPs with HRP from striatal astrocytes. It can be readily adapted to profile CSPs from other cell types or brain regions by using AAVs driven by alternative cell type-specific promoters or by combining conditional AAVs with appropriate Cre driver mouse lines.

In this protocol, we typically generate four biological replicates for proteomic analysis, with each replicate prepared from the striata of three mice. The procedure can be scaled to include additional samples or adapted to isolate CSPs from other cell types, provided that the appropriate mouse lines or AAV tools are available.

We recommend preparing three batches of mice for the following steps. Batch 1: Four animals (four biological replicates) for immunohistochemical characterization of HRP biotinylation. For each animal, half of the tissue serves as the HRP-labeling group and the other half as the non-reaction control (omitting BxxP substrate or H_2_O_2_). Batch 2: Four animals (four biological replicates) for streptavidin blot validation of HRP biotinylation, processed in the same way as Batch 1 (half labeled, half control). Batch 3: Twenty-four animals (four biological replicates) for proteomic analysis. Twelve animals serve as four replicates for the HRP-labeling group, and twelve as four replicates for the non-reaction control. Each replicate consists of pooled tissue from three mice. The total animal number required may vary depending on the sensitivity of the mass spectrometry platform. For the example experiment described here, LC–MS/MS analysis was performed using a Thermo Scientific Orbitrap Fusion Lumos mass spectrometer coupled to a Dionex UltiMate 3000 RPLC-nano system.

### Innovation

Existing approaches to characterize cell-surface proteins (CSPs) are largely restricted to in vitro applications. Traditional methods based on HRP-conjugated antibodies face limited tissue penetration, while biotin ligase–based proximity labeling approaches (such as BioID or TurboID) depend on intracellular ATP and therefore exhibit low efficiency for extracellular labeling in vivo. Genetically encoded, membrane-anchored HRP system uses non-cell-permeable substrate biotin-xx-phenol (BxxP) in acute slices. HRP catalyzes rapid and confined labeling of extracellular proteins at the cell surface, providing superior temporal control and less intracellular background comparing to TurboID. This strategy preserves native cell–cell interfaces and allows selective enrichment of bona fide surface proteins under near-physiological conditions. Compared with previous surface proteomic methods, this HRP-based approach uniquely combines spatial precision, speed, and in situ applicability, enabling the systematic identification of cell type–specific surface proteomes in the brain. It can be broadly applied to dissect intercellular interfaces between neurons, astrocytes, and other brain cell types, providing a powerful platform to investigate molecular mechanisms of cell–cell communication in both healthy and diseased states.

### Institutional permissions

Animal experiments were conducted in accordance with the National Institute of Health Guide for the Care and Use of Laboratory Animals and were approved by the Chancellor’s Animal Research Committee at the University of California, Los Angeles. All mice were housed with food and water available ad libitum in a 12 h light/dark environment. All animals were euthanized during the light cycle, and none were involved in previous studies.

### AAV transduction to express cell-surface HRP in mice

**Timing: 6–8 weeks**

Typically, either of two ways can be used to induce HRP expression in cells of interest: using adeno associated viruses (AAVs) or mouse lines.^8^ This protocol will focus on AAV based expression. The HRP construct contains an N-terminal HA tag and a C-terminal transmembrane (TM) domain from platelet-derived growth factor receptor β (PDGFRβ), which anchors HRP to the extracellular surface of the plasma membrane.

1. Acquire plasmid HRP according to (Table 1 and key resources table) from Addgene or Lead Contact.
2. Prepare AAV Plasmid.
  a. Transform *GfaABC*_*1*_*D* HA-HRP-TM plasmid into competent *E. coli* (Top10 or DH5α).
  b. Grow the transformed bacteria in 300 mL LB medium containing ampicillin at 37°C with shaking for 12–16 h.
  c. Isolate plasmid DNA using an endotoxin-free Maxiprep kit (e.g., Qiagen, Cat. No. 12362).
  d. Verify the plasmid sequence by nanopore sequencing.
3. Generate AAV expressing *GfaABC*_*1*_*D* HA-HRP-TM.
  a. Send Maxi prepped *GfaABC*_*1*_*D* HA-HRP-TM plasmids to a core facility or commercial vendor for AAV production. AAV can also be made in-house if the expertise, resources, and biosafety compliance permit. Use AAV5 for local expression or AAV PHP.eB for whole brain expression.
  b. Aliquot the received viruses into 10 μL portions to minimize freeze–thaw cycles. Store purified AAV5 at −80°C for long-term use (stable for several years). For AAV-PHP.eB, maintain stock at 4°C.

**CRITICAL:** Choose the AAV serotype based on the target cell type. In this protocol, AAV5 was selected because it drives efficient and broad astrocyte transduction.

***Note:*** To adapt the workflow for other cell types, Cre-dependent HRP AAVs can be combined with appropriate Cre driver lines. For astrocytes, Aldh1l1-Cre/ERT2 mice are highly effective.^9^

4. Intracranial injection of AAV5 HRP or retroorbital injection of AAV PHP.eB HRP.
  a. Microinject approximately 10^10^ genome copies (GC) per mouse in a total volume of 500 nL of AAV (2 × 10^13^ GC/mL) intracranially into striatum three weeks before characterization or proteomics. Stock titer may vary by batch/vendor and can be adjusted to achieve the same GC/site at the stated volume.
    **CRITICAL:** For injections into the striatum, we used stereotaxic coordinates of 0.8 mm anterior/posterior, 2.0 mm medial/lateral, and 2.6 mm dorsal/ventral relative to the pial surface. Coordinates for additional brain regions can be obtained from reference atlases such as the Allen Mouse Brain Atlas or Paxinos and Franklin’s Mouse Brain Atlas.
  b. Alternatively, retro-orbitally inject 10^12^ genome copies of PHP.eB HRP AAV per mouse in a total volume of 50 μL saline four weeks before characterization or proteomics.

**Table 1:** Available HRP plasmids

HRP plasmid	AAV stereotype	Targeting cell type
*GfaABC*_*1*_*D* HA*-*HRP-TM	AAV2/5, PHP.eB	Astrocytes
*hSyn1* HA-HRP-TM	AAV2/9, PHP.eB	Neurons
CAG HA-HRP-TM	AAV X1.1	Endothelial cells
FLEX CAG HA-HRP-TM	Various	Cre-dependent

**Figure 1:**
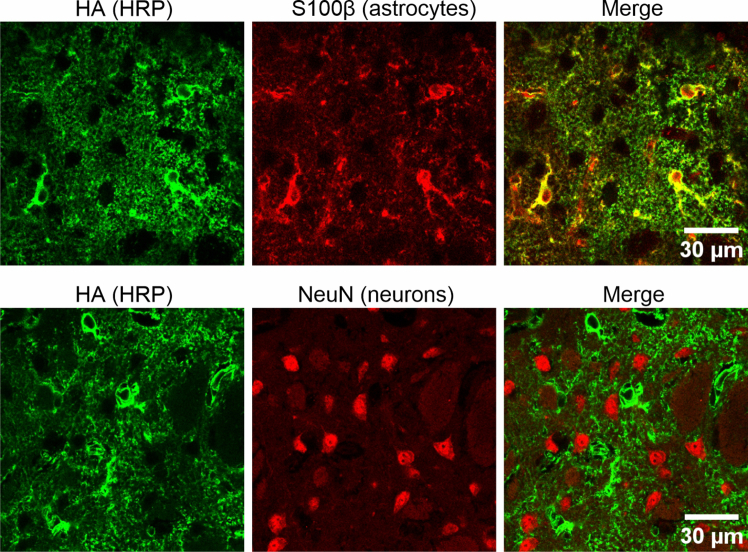
Immunohistochemical validation of HRP expression Example immunohistochemistry images illustrate astrocyte-specific HRP expression, showing overlap with S100β (A) and no colocalization with NeuN (B).

### Test cell specificity of HRP expression

**Timing: 3 weeks**

5. We recommend injecting HRP AAVs into four animals to assess cell-type specificity of HRP expression via immunohistochemistry prior to conducting proximity labeling and proteomic experiments.

***Note:*** This step is especially important when targeting new brain regions or using untested Cre driver lines. Colocalization of HRP within specific brain cell types can be evaluated using anti-HA antibodies in combination with cell-type-specific markers (Figure 1). A list of validated antibodies used in previous experiments is provided in the key resources table.

## Key resources table

**Table:** 

REAGENT or RESOURCE	SOURCE	IDENTIFIER
**Antibodies**		

Rabbit anti-S100β 1:1,000 dilution	Abcam	Cat# Ab52642; RRID: AB_882426
Rabbit anti-NeuN 1:1,000 dilution	Cell Signaling Technology	Cat# 12943; RRID: AB_2630395
Mouse anti-GAPDH 1:5,000 dilution	Invitrogen	Cat# MA5-15738; RRID: AB_10977387
Mouse anti-HA 1:1,000 dilution	BioLegend	Cat# 901514; RRID: AB_2565335
Alexa Fluor 488 goat anti-mouse 1:1,000 dilution	Thermo Scientific	Cat# A11001; RRID: AB_2534069
Alexa Fluor 546 goat anti-rabbit 1:1,000 dilution	Thermo Scientific	Cat# A11035; RRID: AB_2534093
Alexa Fluor 647 goat anti-rabbit 1:1,000 dilution	Thermo Scientific	Cat# A21245; RRID: AB_2535812
Streptavidin-conjugated Alexa 647 1:1,000 dilution	Thermo Scientific	Cat# S21374; RRID: AB_2336066

**Virus strains and bacterial strains**		

AAV2/5 *GfaABC*_*1*_*D* HRP-TM	Vector Builder	N/A
AAV PHP.eB:*GfaABC*_*1*_*D*-HRP-TM	CalTec	N/A
DH5α chemically competent cell	New England Biolabs	Cat# C2987H

**Recombinant DNA**		

*GfaABC*_*1*_*D* HA-HRP-TM	Wu et al.^1^	Addgene ID: 231499
*hSyn1* HA-HRP-TM	Wu et al.^1^	Addgene ID: 231500
CAG HA-HRP-TM	Wu et al.^1^	Addgene ID:231501
FLEX CAG HA-HRP-TM	This study	N/A

**Chemicals, peptides, and recombinant proteins**		

Formalin (buffered 10%)	Fisher Chemical	Cat# SF100-20
Normal goat serum	Vector Laboratories	Cat# S-1000-20
BSA	Sigma-Aldrich	Cat# A8806
Biotin-XX tyramide (“BxxP” in the figures)	APExBIO	Cat# A8012
H_2_O_2_	Sigma-Aldrich	Cat# H1009
Sodium ascorbate	Sigma-Aldrich	Cat# PHR1279
(±)-6-Hydroxy-2,5,7,8-tetramethylchromane-2-carboxylic acid (Trolox)	Sigma-Aldrich	Cat# 238813
Streptavidin magnetic beads	Thermo Scientific	Cat# 88816
Halt Protease Inhibitor Cocktail (100×)	Thermo Scientific	Cat# 78429
DTT	Sigma-Aldrich	Cat# D0632-1G
Sodium azide	Sigma-Aldrich	Cat# S2002-25G
Sodium chloride	Sigma-Aldrich	Cat# S9888-500G
Sodium dodecyl sulfate	Sigma-Aldrich	Cat# 436143
Sodium carbonate	Sigma-Aldrich	Cat# 223530
Sodium bicarbonate	Sigma-Aldrich	Cat# S6014
Potassium chloride	Sigma-Aldrich	Cat# P3911-500G
Tris-HCI, pH 8.0	Thermo Scientific	Cat# 15568025
Tris-HCI, pH 7.5	Thermo Scientific	Cat# 15567027
Urea	Sigma-Aldrich	Cat# 31K0038
Mini-PROTEAN TGX Stain-Free Protein Gels	Bio-Rad	Cat# 4568034
2× Laemmli Sample Buffer	Bio-Rad	Cat# 1610737
UltraPure DNase/RNase-Free Distilled Water	Thermo Scientific	Cat# 10977015
Trypsin, Mass spectrometry grade	Promega	Cat# V5113
Lysyl Endopeptidase, Mass spectrometry grade	Fujifilm Wako	N/A
Streptavidin–horseradish peroxidase	Thermo Scientific	Cat# S911

**Critical commercial assays**		

Pierce BCA Protein Assay	Thermo Scientific	Cat# 32106
Pierce ECL Western Blotting Substrate	Thermo Scientific	Cat # 34580

**Experimental models: Organisms/strains**		

Male and female C57Bl/6NTac mice (8–10 weeks)	Taconic	Stock# B6; RRID:IMSR_TAC:b6

**Software and algorithms**		

MaxQuant 2.0.3.0	https://maxquant.org	RRID:SCR_014485
artMS R package	http://artms.org/	https://bioconductor.org/packages/artMS

**Other**		

Tube rotator	Miltenyi Biotec	Cat# 130-090-753
Magnetic stand	Invitrogen	Cat# 12321D
RNAse/DNAse-free 1.5 mL tubes	Axygen	Cat# MCT-185-C
Petri dish for dissection	Brand	Cat# 455742
Dissection microscope	Leica	Cat# S9 E Stereomicroscope
Surgical tools for dissection	FST	Cat# 11210-20, 11252-11271-3000
Dounce homogenizer 2 mL	Kimble	Cat# 885303-002
Pestle A	Kimble	Cat# 885301-002
Pestle B	Kimble	Cat# 885302-002
Tube rotator	Invitrogen	Cat# 88881001
DynaMag-2 Magnet	Invitrogen	Cat# 12321D
Sonicator	Qsonica	Q800R3
GelDoc Go Gel Imaging System	Bio-Rad	N/A
Optima TLX Ultracentrifuge	Beckman	N/A
TLA 120.2 rotor	Beckman	N/A
Amersham Imager 680	General Electric	N/A
Orbitrap Fusion Lumos Tribrid mass spectrometer	Thermo Fisher Scientific	N/A
Dionex UltiMate 3000 RPLCnano system	Thermo Fisher Scientific	N/A

## Materials and equipment

**Table:** Slicing buffer

Chemicals	Final concentration (mM)	Add to 500 mL
Sucrose	194	33.2 g
NaCl	30	0.88 g
KCl	4.5	0.17 g
NaH_2_PO_4_	1.2	0.07 g
NaHCO_3_	26	1.09 g
D-glucose	10	0.9 g
MgCl_2_	1	0.5 mL from 1 M stock

Store at 4°C for up to one week. Make 80 mL/animal. Must be bubbled with 95% O_2_ and 5% CO_2_ for at least 30 min before use.

**Table:** Artificial cerebrospinal fluid

Chemicals	Final concentration (mM)	Add to 1 L
CaCl_2_	2	2 mL from 1 M stock
MgCl_2_	1	1 mL from 1 M stock
KCl	4.5	0.34 g
NaH_2_PO_4_	1.2	0.14 g
NaHCO_3_	26	2.18 g
D-glucose	10	1.8 g
NaCl	124	7.25 g

Make fresh on the day of use and keep at 4°C. Add CaCl_2_ and MgCl_2_ after dissolving other chemicals to avoid precipitation.

**Table:** High SDS (1%) RIPA buffer

Reagent	Final concentration	Stock solution	Add to 5 mL
NaCl	150 mM	1 M	750 μL
Triton-X	1 %	20 %	250 μL
Na Deoxycholate	0.5 %	10 %	250 μL
SDS	1 %	10 %	500 μL
Tris pH 8.0	50 mM	1 M	250 μL
Protease inhibitor	1×	100×	50 μL
UltraPure water	N/A	N/A	to 5 mL

Store the buffer at 4°C for up to 6 months. Always add protease inhibitor freshly before use. Store the stock solutions at room temperature for up to 6 months, except the protease inhibitor, which should be stored at 4°C.

**Table:** RIPA buffer

Reagent	Final concentration	Stock solution	Add to 10 mL
NaCl	150 mM	1 M	1.5 mL
Triton-X	1 %	20 %	500 μL
Na Deoxycholate	0.5 %	10 %	500 μL
SDS	0.2 %	10 %	200 μL
Tris pH 8.0	50 mM	1 M	500 μL
Protease Inhibitor	1×	100×	100 μL
UltraPure water	N/A	N/A	to 10 mL

Store at 4°C for up to 6 months. Always add protease inhibitor freshly before use.

**Table:** SDS-free RIPA buffer

Reagent	Final concentration	Stock solution	Add to 15 mL
NaCl	150 mM	1 M	2.25 mL
Triton-X	1 %	20 %	750 μL
Na Deoxycholate	0.5 %	10 %	750 μL
Tris pH 8.0	50 mM	1 M	750 μL
Protease Inhibitor	1×	100×	150 μL
UltraPure water	1×	N/A	to 15 mL

Store at 4°C for up to 6 months. Always add protease inhibitor freshly before use.

**Table:** Quench buffer

Chemicals	Final concentration (mM)	mg/20 mL
Trolox	5	25.04
Sodium ascorbate	10	39.64

Prepare fresh on the day of use and keep on ice. Dissolve in ACSF with vigorous shaking; incubation at 37°C can be used to enhance solubility.

**Table:** BxxP substrate

Chemicals	Final concentration	Stock	Add to 10 mL
Biotin-XX tyramide	100 μM	10 mM	100 μL

Prepare fresh on the day of use by diluting in ACSF to a final concentration of 100 μM. The stock solution is dissolved in ACSF with extensive vortex and sonication, and can be stored at −20°C for up to 1 year.

## Step-by-step method details

### Brain slicing and biotinylation of cell-surface proteins in mice

**Timing: 1–4 days**

This step enables selective biotinylation of cell-surface proteins (CSPs) from defined cell types in freshly prepared mouse brain slices. Incubation with the non-permeable substrate BxxP and brief H_2_O_2_ exposure catalyzes HRP-dependent biotinylation confined to the extracellular surface. The resulting labeled slices provide high-specificity samples for downstream validation or proteomic analysis of native cell-surface proteomes.

**Figure 2:**
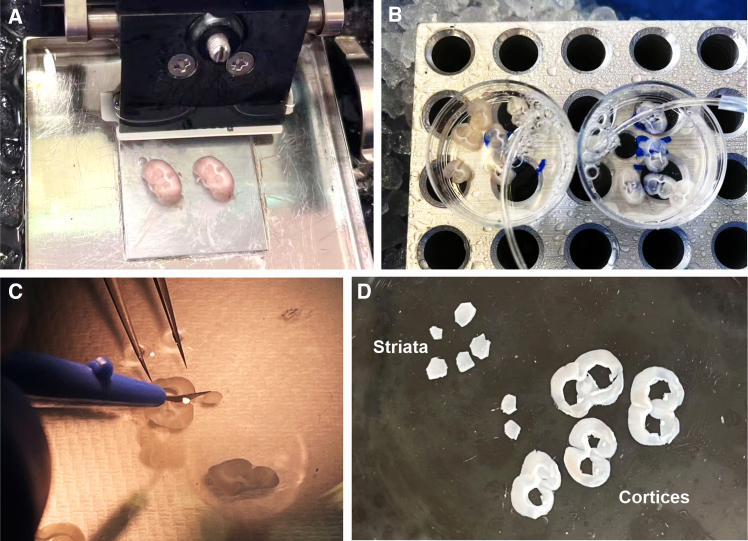
Pictures illustrating striata dissection (A) Brains are glued upwards to slice striatum in coronal sections. (B) Incubation of brain slices with BxxP in ACSF with bubbling. (C) Dissect striatum under endoscope. (D) Dissected striata and left over cortices.

1. Brain slicing.
  ***Note:*** Follow standard brain slice protocols for electrophysiology, slice @ 300 μm thickness. The following is the protocol from our lab with DSK-Zero 1 slicer.
  a. Bubble (oxygenate) slicing buffer (95% O_2_ + 5% CO_2_) on ice for 30 min using a fine sinter.
  b. Place clean surgical instruments and vibratome stage on ice.
  c. Prepare vibratome before dissection:
    i. Fill ice and cold water in the chamber.
    ii. Mount the blade, make sure the blade cuts horizontally.
  d. Mouse brain isolation.
    i. Euthanize the mouse according to institutional guidelines.
    ii. After euthanasia, decapitate the animal using sharp surgical scissors.
    iii. With sterile surgical instruments, carefully remove the brain and transfer it immediately into a 50 mL beaker containing ice-cold slicing buffer.
      **CRITICAL:** We anesthetize mice in a bell jar containing an isoflurane-soaked swab, ensuring that the swab does not contact the skin. Confirm loss of reflexes and absence of breathing before proceeding (e.g., by toe pinch). Once euthanasia is complete, carry out all subsequent steps on ice.
  e. Dry the cooled vibratome stage with filter paper and apply a small dot of glue on the center of the stage.
  f. Remove cerebellum with ice-cold blade.
  g. Gently lower the rest of the brain onto the glue with the olfactory bulb facing up.
  h. Hold the brain in place for about 10–20 seconds until the glue sets. Avoid applying pressure.
  i. Place the stage in the chamber and secure it.
  j. Fill the stage with cold and oxygenated slicing buffer.
  k. Slice at 500 μm sections until striatum appears, switch to 300 μm thickness (Figure 2A).
    ***Note:*** Typically, 4–5 coronal brain slices containing the striatum can be collected.
2. Biotinylation reaction.
  a. Move each brain slice with a brush after sectioning immediately into a 35 mm small dish containing 3 mL 100 μM BxxP-in ACSF, incubate on ice for 1 hour, with 95% O_2_ 5% CO_2_ oxygenation (Figure 2B).
  b. Move the dish to room temperature, add 3 μL H_2_O_2_ (3 % stock) for the 5-minute labeling.
    **CRITICAL:** Make sure the H_2_O_2_ solution is not expired. Protect the stock from light and store @ 4°C.
  c. Quench the reaction immediately by five thorough washes with cold quench buffer.
  d. Remove the quench solution, add cold oxygenated ACSF.
  e. For IHC characterization, fix the slices in 10% Formalin in PBS @ 4°C overnight.
  f. For streptavidin blot and proteomics, dissect striata in ACSF under stereo microscope (Figures 2C and 2D), collect striata into Eppendorf tubes, snap freeze in liquid nitrogen and store at −80°C.
    **CRITICAL:** We recommend performing labeling and control reactions in parallel. Control reactions can omit BxxP incubation or omit H_2_O_2_ treatment.
    ***Note:*** For IHC and streptavidin blot, typically we slice two brains at a time, and prepare four small dishes with oxygenation. Divide the slices from each animal equally into two dishes, one for labeling condition, one for non-labeling control condition. Thus, one round of slicing will generate two replicates for each condition.
    ***Note:*** For proteomics, typically we slice six brains at a time, and prepare two small dishes with oxygenation. Pool the slices from three animals into each dish, one for labeling condition, one for non-labeling control condition. Thus, one round of slicing will generate one replicate (pooled from three animals) for each condition. 24 animals need 4 rounds of slicing and generate 4 replicates for each condition.
    **Pause point:** For IHC, we normally perform staining the next day. The fixed slices can also be short-term stored at 4°C in PBS + 0.02–0.05% NaN_3_ for 2–4 weeks. For streptavidin blot and proteomics, sample processing can continue immediately or be performed on another day. Process within ≤3 months when possible, frozen samples store up to 12 months. Avoid auto-defrost freezers and minimize time at room temperature during handling.

### Characterization of HRP labeling by IHC

**Timing: 3 days**

This step accomplishes visualization and validation of HRP-mediated biotinylation at the cellular level. The resulting confocal images verify successful HRP expression and extracellular biotinylation, providing critical quality control before proceeding to biochemical enrichment and proteomic analysis.

Use batch 1 animals for IHC. The flowing steps continue after brain slice fixation (step 2e).

3. Primary antibody staining (HA, S100β).
  a. Wash brain sections three times in PBS containing 2% Triton X-100, 5 min each wash.
  b. Block sections for 1 h at room temperature in PBS containing 1% Triton X-100 supplemented with 10% normal goat serum (NGS) while gently agitating.
  c. Incubate slices with HA and S100β primary antibodies (1:1000 in PBS containing 0.4% Triton X-100) at 4°C for 2–3 days with continuous agitation.
4. Streptavidin staining.
  a. Wash the sections 3 times in PBS with 0.4% Triton-X for 10 min each.
  b. Incubate 2 hrs at RT with secondary antibodies and streptavidin conjugated Alexa 647 (1:500) diluted in PBS with 0.4% Triton-X.
5. Mounting and imaging.
  a. Rinse the sections 3 times in PBS for 10 min each.
  b. Mount samples on glass slides using Fluoromount-G.
  c. Image the slides with a confocal microscope.

### Characterization of HRP labeling by streptavidin blot

**Timing: 2 days**

This step accomplishes biochemical validation of HRP-mediated biotinylation through detection of biotinylated proteins in tissue lysates.

Use batch 2 animals for streptavidin blots. The following steps continue after striata dissection (step 2f).

6. Lysis of the tissue.
  a. Dounce homogenize each striatal samples with 300 μL high-SDS (1%) RIPA buffer at least 15 times.
  b. Collect 300 μl homogenate into a sonication tube.
  c. Sonicate for 5 mins (30 seconds on, 30 seconds off) at 60% duty cycle (QSONICA, Q800R3 Sonicator).
    ***Note:*** We use a QSonica Q800R3 Bath Sonicator as the volume is small, standard probe sonicators could also work if the probe fits into a 1.5 mL tube.
  d. Heat the samples at 95°C for 5 minutes.
  e. Transfer each sample into a 2 mL tube, add 1200 μL SDS-free RIPA buffer into each tube, rotate at 4°C for 1 hr.
  f. Transfer samples into ultra centrifuge tubes. Ultra centrifuge at 50 k rpm for 45 minutes at 4°C.
  g. Collect and keep supernatant (lysate) on ice.
  h. Measure the protein concentration of lysate using bicinchoninic acid (BCA) assay.
7. Streptavidin blot.
  a. Prepare samples in 2× Laemmli buffer supplemented with β-mercaptoethanol (following the manufacturer’s protocol), then boil the samples for 10 min at 95°C.
  b. Load ∼30 μg of protein per lane on a 10% precast polyacrylamide gel and run the gel until the dye front reaches the bottom.
  c. Image total loaded proteins with Bio-Rad Geldoc imager to verify that protein loading is comparable between samples.
    ***Note:*** We use a Bio-Rad mini Protean Stain-free Gel system. Alternatively, total proteins can be visualized after transfer with Ponceau S.
  d. Transfer the proteins to a nitrocellulose membrane.
    ***Note:*** Transfer efficiency can be checked with Ponceau S; destain with PBST before proceeding.
  e. Following transfer, block membranes for 1 h at room temperature with 5% BSA in PBST while shaking.
  f. Prepare the streptavidin–HRP probe (0.3 μg/mL in 5% BSA/PBST) and incubate membranes for 2 h at room temperature with agitation.
  g. Wash three times in PBST, 7 min each, with shaking.
  h. Develop membranes using Pierce ECL substrate for ∼1 min.
  i. Acquire chemiluminescent signal with an imaging system.
    ***Note:*** We used a GE Amersham Imager 680 in chemiluminescence mode. Equivalent systems such as ChemiDoc MP (Bio-Rad Laboratories) may be substituted.
  j. After imaging, wash membranes three times in PBST for 7 min each with shaking.
  k. Incubate overnight at 4°C with gentle agitation in a primary antibody cocktail prepared in 5% BSA/PBST.
    **CRITICAL:** GAPDH is used here as a loading control, but alternatives such as β-actin can also be used. For detecting HA-tagged HRP, mouse anti-HA (BioLegend, Cat. No. 901514) is effective at 1:1000.
  l. Wash membranes three times in PBST for 7 min each with shaking.
  m. Incubate for 1 h at room temperature with fluorophore-conjugated secondary antibodies diluted in 5% BSA/PBST.
  n. Wash membranes three times in PBST for 7 min each with shaking.
  o. Acquire fluorescent images using an appropriate imaging system.
    ***Note:*** In our setup, Alexa-conjugated secondary antibodies (1:2000) were imaged on a GE Amersham Imager 600 in fluorescence mode.
    **CRITICAL:** Use the streptavidin–HRP signal to assess HRP-dependent biotinylation relative to negative controls. Endogenous biotinylated proteins (∼130 kDa and 72–75 kDa, mitochondrial carboxylases) should appear consistently. Normalize signals to the selected loading control.

### Enrichment of biotinylated CSPs

**Timing: 2 days**

This step accomplishes the selective enrichment of biotinylated cell-surface proteins (CSPs) from tissue lysates for downstream proteomic analysis.

After confirming HRP labeling with IHC and streptavidin blot, use batch 3 animals for proteomics.

***Note:*** The following steps continue after step 2f.

8. Lysis of the tissue as in step 6, adjust the volume of each sample with RIPA buffer to reach the same concentration.
9. Wash streptavidin magnetic beads twice with 1 mL RIPA buffer. Use ∼50 μL beads per tube for 300 μg protein. Save remaining lysate as input for streptavidin blot analysis (store at −20°C).
10. Incubate beads with post-ultracentrifugation lysates overnight at 4°C on a rotator.
11. Collect beads with a magnetic rack and transfer 20 μL of the supernatant to fresh tubes for streptavidin blot analysis (flow-through).
12. Wash beads twice with 1 mL RIPA buffer for 2 min @ RT.
13. Rinse once with 1 mL 1 M KCl for 2 min @RT.
14. Rinse once with 1 mL 0.1 M Na_2_CO_3_ for ∼10 s.
15. Rinse once with 2 M urea in 10 mM Tris-HCl (pH 8.0, 1 mL) for ∼10 s.

**CRITICAL:** Minimize bead exposure to Na_2_CO_3_ or urea; prolonged (>10 min) contact can cause bead denaturation and aggregation.

16. Wash bead-bound proteins with 400 μL 50 mM Tris-HCl (pH 7.5).
17. Follow with two washes using 300 μL 2 M urea in 50 mM Tris-HCl (pH 7.5). Adjust wash volume if working with larger bead quantities.
18. Take 5 μL beads from each sample for verification.
19. Keep the rest of the beads in 50 mM Tris pH 7.5 @ 4°C, follow with proteomic sample processing and mass spectrometry session (see below).

**CRITICAL:** Do not freeze beads. Proceed to on beads digestion within 24 h.

20. Elute the enriched protein from the 5 μL bead samples by boiling in 20 μL 2× protein loading buffer with 2 mM biotin and 20 mM DTT at 95°C for 10 min.
21. Store the eluate @ −20°C for verification of enrichment after pull-down.
22. Perform a streptavidin blot to visualize the biotinylated proteins from collected eluates. Experimental samples should show stronger enrichment compared to negative controls (troubleshooting, problem 1 and 2).

***Note:*** To optimize bead usage prior to proteomics, elute material from test samples by boiling in 2× protein loading buffer with 2 mM biotin and 20 mM DTT (10 min, 95°C). Run input, flow-through, and eluate samples on a 10% SDS-PAGE gel and probe with streptavidin–HRP (see Step 7). Confirm depletion of biotinylated proteins in the flow-through and their enrichment in the eluate. If biotinylated signal remains in flow-through, increase bead volume (e.g., 1.5–2×) and repeat until the signal is near background.

### Proteomic sample processing and mass spectrometry

**Timing: 2–4 days**

This step accomplishes proteolytic digestion and preparation of digested biotinylated cell-surface proteins for quantitative mass spectrometry analysis, enabling quantitative identification of cell-surface proteomes across experimental conditions.

23. Resuspend the beads in 2 M urea, 50 mM Tris-Cl, pH 8.0 and reduce the protein complexes bound to streptavidin beads with 5 mM tris(2-carboxyethyl) phosphine. Incubate the beads on shaking at 25°C for 30 minutes.
24. Alkylate the reduced protein complexes bound to streptavidin beads using 10 mM iodoacetamide. Incubate the beads on shaking at 25°C for 30 minutes. Protect from light.

**CRITICAL:** Iodoacetomide is light sensitive, hence carry out the alkylation step in dark.

25. Add trypsin and lysC proteases at 1:50 and 1:100 enzyme to protein ratio respectively. Perform proteolytic digestion at 37°C overnight.
26. Quench the digestion reaction by addition of formic acid to a final concentration of 5%.
27. Remove the tryptic digest sample from the beads using a magnetic rack.
28. Desalt the tryptic digest using C18 desalting tips according to the manufacturer’s protocol.
29. Dry the desalted peptides using vacuum centrifugation and resuspend in 5% formic acid.
30. Analyze the desalted peptides by LC-MS/MS (troubleshooting, problem 3).

***Note:*** This protocol generates samples that can be analyzed with a wide range of LC/MS-MS and analysis pipelines. Experimental details regarding liquid chromatography, mass spectrometry, and data analysis will vary widely based on the exact analytical platform employed. Users can refer to Wu et al, 2025^1^ for details regarding our exact experimental setup but should implement workflows based on their own individual setups. As a reference, the dried desalted peptides were subjected to online fractionation on a 25 cm long and 75 μm inner diameter fritted fused silica column using a gradient of increasing acetonitrile. The eluted peptides were ionized using electrospray ionization before injection into Orbitrap Fusion Lumos mass spectrometer being operated in a data-dependent acquisition mode. The raw data were analyzed using the MaxQuant bioinformatics pipeline. More details about the settings of LC-MS/MS and bioinformatics pipelines are provided in Table 2.

**Table 2:** Mass spectrometry analysis using Orbitrap Fusion Lumos mass spectrometer on the data-dependent acquisition mode

Column specifications	Fritted fused silica 25cm (length) X75μm (inner diameter)
	Dimensions- 25cm (length) X75mm (inner diameter)
	Material- bulk 1.9 μm ReproSil-Pur beads with 120 Å pore size
Peptide fractionation	System- Dionex ultimate 3000
	Gradient specifications- 5%–80% acetonitrile
	Flow rate- 200nL/min
Mass spectrometry	System- Orbitrap Fusion Lumos
	Mode of operation- Data dependent acquisition (DDA)
	MS1 scans at 120,000 resolution
	MS2 scans at 15000 resolution
Bioinformatics	MaxQuant
	Peptide identification- Andromeda
	Reference proteome- Mus musculus (UP000000589)
	Search parameters- 2 maximum missed cleavages
	1% FDR at peptide and protein level
	Label free quantification (LFQ), minimum ratio count 1
	mass tolerance threshold for precursor ion set to 20ppm
	mass tolerance threshold for fragment ion set to 4.5ppm
Statistics	Analytical R tools for Mass Spectrometry (artMS)

## Expected outcomes

**Figure 3:**
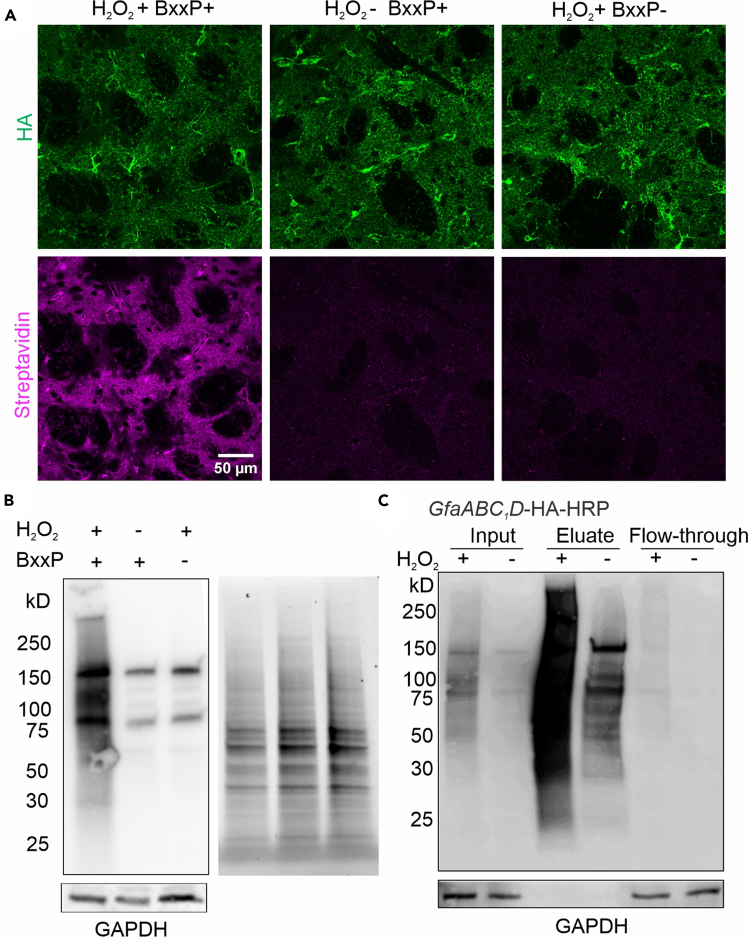
Example data validating HRP biotinylation and enrichment of biotinylated proteins (A) Confocal images of striatal Astro HRP (green). Biotinylated astrocytic CSPs are indicated by streptavidin IHC (magenta). (B) Left, streptavidin blot showing biotinylation of astrocytic CSPs by HRP from indicated conditions. GAPDH was the loading control. Right, stain-free blot (with Bio-Rad stain-free precast PAGE gel) before membrane transfer showing total amount of proteins loaded corresponded to streptavidin blot on the left. (C) Streptavidin blot of biotinylated astrocytic cell-surface proteins during different steps of the pull-down. Notice the strong biotinylation intensity observed from the eluate of H_2_O_2_ treated samples compared with input and flow-through, indicating enrichment of biotinylated proteins within the eluate. Figure reprinted and adapted from Wu et al., 2025.

Correctly expressed HRP should colocalize with markers enriched in the targeted cell type *in vivo*. For example, Figure 1 shows confocal images of astrocyte-specific HRP expression. HRP-driven biotinylation requires both the BxxP substrate and H_2_O_2_ as cofactors; examples are shown in confocal imaging and streptavidin blotting (Figures 3A and 3B). In our hands, AAV5-Astro-HRP locally delivered to the striatum resulted in specific expression in approximately 90% of striatal astrocytes, while AAV-PHP.eB-Astro-HRP delivered retro-orbitally to the whole brain achieved specific expression in roughly 80% of striatal astrocytes.

**Figure 4:**
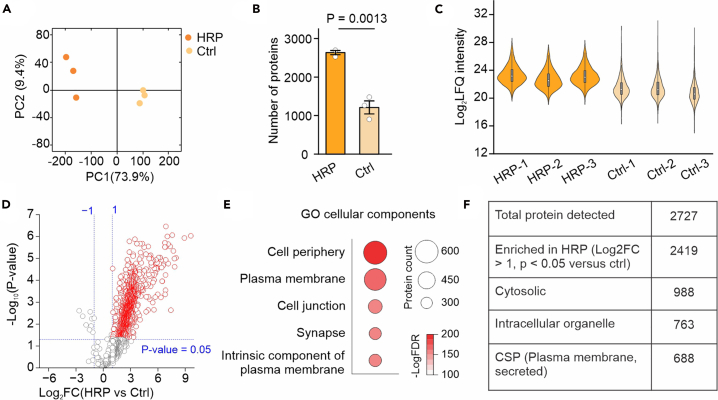
Example data validating HRP proteomics (A) Principal component analysis (PCA) showing clear separation between HRP-labeled samples (with H_2_O_2_) and control samples (without H_2_O_2_). (B) Bar graphs of total protein numbers detected in each of the HRP samples and control samples. Error bars indicate standard error. Student's t-test. (C) Violin plots of LFQ intensity distributions across all proteomic samples. (D) Volcano plots indicate the CSPs enrichment in HRP condition relative to control condition. (E) Top five cellular compartment terms (−logFDR) from astrocytic CSPs with GO analysis. (F) Table reporting the number of proteins of proteins detected for each indicated category with astrocyte cell-surface HRP. Figure reprinted and adapted from Wu et al., 2025.

The success of proteomic experiments depends on efficient enrichment of biotinylated proteins captured by streptavidin beads. This can be evaluated by blotting the flow-through and eluate fractions (Figure 3C). Data quality may be further assessed using PCA plots of peptide or protein intensities, where labeled samples are expected to segregate from non-labeled controls along the first principal component (Figure 4A). Quality-control metrics generated by artMS (qcBasic or qcExtended) should also indicate a higher number of detected proteins and greater Log_2_ LFQ intensity in the HRP group (Figures 4B and 4C).

Proteins significantly enriched in HRP-labeled samples relative to controls can be identified using artMS integrated with MSstats, applying thresholds of p < 0.05 (or FDR-adjusted < 0.05) and log_2_ fold change > 1. A representative volcano plot should illustrate significant enrichment in HRP-labeled samples (Figure 4D). Known markers of the targeted cell type (such as Slc1a2, Aqp4, and Gja1 for astrocytes) should be detected among the enriched proteins. GO-Cellular Component enrichment analysis of these proteins should reveal overrepresentation of cell-surface-related terms (Figure 4E). It is important to note that the enriched biotinylated proteins may also include a substantial fraction of intracellular proteins, either due to technical limitations of the labeling procedure or underlying biological factors. These can be filtered out using in silico bioinformatic approaches. An example summary table listing total proteins, intracellular proteins, and CSPs is shown in Figure 4F.

In addition to plasma membrane proteins of the targeted cell type, this approach is expected to capture cell-surface proteins secreted by or anchored on neighboring, non-targeted cells that are in close physical contact. Such cross-cell labeling reflects genuine cell–cell interface proximity rather than nonspecific diffusion. In our affiliated Neuron paper, we validated this outcome in vivo using proximity ligation assays (PLA) and immunohistochemistry (IHC), confirming more than ten CSPs originating from distinct cell types at astrocyte–neuron interfaces. We also used publicly available single-cell RNA sequencing (scRNA-seq) databases to predict the cellular origin of these CSPs. Similar validations and analysis can be performed to confirm spatial localization and cellular origin of candidate proteins identified by users.

## Quantification and statistical analysis

The data generated using data-dependent acquisition scheme on Orbitrap Fusion Lumos Tribrid instrument is analyzed on MaxQuant bioinformatic pipeline. Use MaxQuant with the integrated Andromeda search engine to identify peptides against the Mus musculus Uniprot reference database (UP000000589). Allow up to two missed cleavages and set the false discovery rate for both peptides and proteins to 1%. Enable label-free quantification (LFQ) with a minimum ratio count of one. Set the parent ion tolerance to 20 ppm and the fragment ion tolerance to 4.5 ppm. Process the MaxQuant output files in with the R package artMS (http://artms.org/) to assess differential protein enrichment. Apply global normalization across all MS runs with built-in MSstats functions to equalize median intensity and select Tukey’s median polish (TMP) as the summary method. Censor missing values and include in the analysis only proteins detected in at least two out of three biological replicates for one condition.

H_2_O_2_+ (HRP labeling) and H_2_O_2_- (non-labeling contrl) groups need to be analyzed for each experiment so that H_2_O_2_–dependent enrichment could be determined independently for each set of mice (log2(FC) of LFQ > 1 and p-value < 0.05 versus H_2_O_2_- controls). Perform cellular component enrichment analysis use the STRING database to evaluate whether the identified proteins were enriched for cell-surface components (GO-CC). Similar analyses can also be conducted using other tools such as Enricher or PANTHER.

To generate a list of CSPs with high confidence, enriched proteins should then be searched for the annotation of subcellular localization in the UniProt database. Proteins with plasma membrane related (extracellular space, cell surface, plasma membrane, secreted, postsynaptic membrane, presynaptic membrane etc.) annotations are curated CSPs. Proteins with either nucleus, mitochondrion, or cytoplasm annotation but without the plasma membrane annotation are classified as non-CSPs. Caution should be exercised with non-CSPs, as this group may still contain previously unrecognized CSPs. To assign the cellular origin of CSPs, one can leverage publicly available scRNA-seq datasets. An example code file demonstrating how to do CSP-mapping with a reanalyzed striatal sing-cell dataset from Allen institute^10^ is provided in Data S1.

## Limitations

While HRP-based approaches have been applied to profile CSPs in the mouse brain, some constraints remain. Biotinylation is restricted to electron-rich residues (histidine, tryptophan, tyrosine), so secreted proteins or peptides lacking these residues may be underrepresented. In addition, detecting small or low-abundance proteins remains technically challenging. Second, because the membrane-impermeant substrate (BxxP) does not cross the blood–brain barrier, tissue must be acutely sliced for *ex vivo* incubation. Acute slicing inevitably creates a superficial damaged rim that can transiently compromise plasma membranes and disrupt neuronal processes at the surface of slices, potentially exposing intracellular compartments to extracellular HRP during labeling. Third, HRP requires H_2_O_2_ as the activator, which can display cytotoxicity. In the future, new proximity-labeling enzymes that bypass the requirement of extracellular ATP or H_2_O_2_ could be utilized.^11^^,^^12^ Fourth, our use of HRP is based on estimates that proximity-dependent biotinylation occurs over tens of nanometers.^13^ However, such distances have not been measured in complex tissues where HRP proximity, reactive radical diffusion, and location and concentration of quenchers are different. Thus, the labelling radius of HRP could be up to ∼300 nm.^14^ This range (10–300 nm) allows detection of proteins not only from the HRP-targeted cell membrane but also from neighboring cells that form close contacts. Prediction of cellular origins for CSPs can be done with publicly available RNAseq data of appropriate brain regions. However, as with all multiomic data, further validations will be needed on a case-by-case basis, and mapping will be aided by increased depth of RNAseq. Inherently, CSP mapping alone does not determine whether a candidate protein interacts directly with the targeted cell or associates indirectly. Additional methods (i.e., pull-down assay, FRET-microscopy and coimmunoprecipitation) should be used to further validate interactions.

## Troubleshooting

### Problem 1

Poor enrichment of biotinylated proteins (replated to step 22 and Figures 3B and 3C).

### Potential solution

This may result from low or absent HRP expression. Increase expression by microinjecting a larger volume or higher titer of AAV into the target region and confirm injection success. Inefficient biotinylation is another possibility; verify that H_2_O_2_ is fresh and active.

### Problem 2

High background biotinylation signal observed in negative control samples (replated to step 22 and Figures 3B and 3C).

### Potential solution

Background often arises from nonspecific protein binding to streptavidin beads. Improve stringency by extending or adding wash steps and reduce bead volume if necessary. To determine the minimal bead amount that still captures the majority of biotinylated proteins, compare flow-through and bead-bound fractions by western blot and select the bead volume at which the flow-through is depleted of signal.

### Problem 3

Low yield of biotinylated proteins (replated to step 30 and Figure 4).

### Potential solution

Yields may be reduced if the starting material is insufficient; increase the amount of input protein. Another cause can be underuse of streptavidin beads; adjust bead volume upward as needed. Finally, incomplete elution during on-bead digestion should be considered; check the pH of the digestion buffer and increase the trypsin concentration if required.

### Problem 4

LC-MS results do not include proteins previously reported as astrocytic or from the expected cell type (related to expected outcomes).

### Potential solution

Even if known markers are absent, the identified candidates may still be valid, as no single approach can capture the entire CSP repertoire. Increase reliability by preparing and analyzing multiple independent samples.

## Resource availability

### Lead contact

Further information and requests for resources and reagents should be directed to and will be fulfilled by the lead contact, Dr. Ling Wu (liwu@mednet.ucla.edu).

### Technical contact

Questions about the technical specifics of performing the protocol should be directed to the technical contact, Dr. Ling Wu (liwu@mednet.ucla.edu).

### Materials availability

This study did not generate new unique materials, except for the FLEX-CAG-HA-HRP-TM plasmid, which can be obtained by contacting the lead contact.

### Data and code availability

This study did not generate datasets or code.

## Acknowledgments

This work, B.S.K., L.W., V.P., and J.A.W. were supported by the National Institutes of Health (R01DA047444, R35NS111583, and R01AG0759655), an Allen Distinguished Investigator Award, a Paul G. Allen Frontiers Group advised grant of the Paul G. Allen Family Foundation, and the Ressler Family Foundation (to B.S.K.). B.S.K. was also partly supported by MH134926 and by the Eleanor I. Leslie Chair in Neuroscience. L.W. was partly supported by a Janssen Research and Development, LLC award (to B.S.K.). We thank David Pan for helping with the code for cell type mapping.

## Author contributions

Conceptualization, B.S.K. and L.W.; experiment and methodology, L.W. and V.P.; writing – original draft, L.W.; writing – review and editing, L.W., V.P., B.S.K., and J.A.W.

## Declaration of interests

The authors declare no competing interests.

## Supplemental information

Data S1. Python notebook file containing code to perform CSP-mapping, related to Quantification and statistical analysis
